# Real-time deep learning semantic segmentation during intra-operative surgery for 3D augmented reality assistance

**DOI:** 10.1007/s11548-021-02432-y

**Published:** 2021-06-24

**Authors:** Leonardo Tanzi, Pietro Piazzolla, Francesco Porpiglia, Enrico Vezzetti

**Affiliations:** 1 Department of Management, Production and Design Engineering, Polytechnic University of Turin, Turin, Italy; 2 Division of Urology, Department of Oncology, School of Medicine, University of Turin, Turin, Italy

**Keywords:** Deep learning, Neural network, Semantic segmentation, Intra-operative

## Abstract

**Purpose:**

The current study aimed to propose a Deep Learning (DL) and Augmented Reality (AR) based solution for a in-vivo robot-assisted radical prostatectomy (RARP), to improve the precision of a published work from our group. We implemented a two-steps automatic system to align a 3D virtual ad-hoc model of a patient’s organ with its 2D endoscopic image, to assist surgeons during the procedure.

**Methods:**

This approach was carried out using a Convolutional Neural Network (CNN) based structure for semantic segmentation and a subsequent elaboration of the obtained output, which produced the needed parameters for attaching the 3D model. We used a dataset obtained from 5 endoscopic videos (*A, B, C, D, E*), selected and tagged by our team’s specialists. We then evaluated the most performing couple of segmentation architecture and neural network and tested the overlay performances.

**Results:**

U-Net stood out as the most effecting architectures for segmentation. ResNet and MobileNet obtained similar Intersection over Unit (IoU) results but MobileNet was able to elaborate almost twice operations per seconds. This segmentation technique outperformed the results from the former work, obtaining an average IoU for the catheter of 0.894 (*σ* = 0.076) compared to 0.339 (*σ* = 0.195). This modifications lead to an improvement also in the 3D overlay performances, in particular in the Euclidean Distance between the predicted and actual model’s anchor point, from 12.569 (*σ*= 4.456) to 4.160 (*σ* = 1.448) and in the Geodesic Distance between the predicted and actual model’s rotations, from 0.266 (*σ* = 0.131) to 0.169 (*σ* = 0.073).

**Conclusion:**

This work is a further step through the adoption of DL and AR in the surgery domain. In future works, we will overcome the limits of this approach and finally improve every step of the surgical procedure.

## Introduction

In recent decades, technology has helped several medical procedures to massively improve [1]; in particular, great progresses have been achieved with Deep Learning (DL) [2] paradigms. Recently, DL has acquired a fundamental role in the medical environment, and many different remarkable applications have been implemented, from orthopedics [3, 4] to oncology [5] and many others [6]. The same enhancement was brought in by Augmented Reality (AR), in particular in surgery. Neurosurgery has been the first medical specialty in which AR was introduced to assist the surgeon [7], followed by orthopedics [8], dental surgery [9] and many more. Surgery, in fact, is one of the main subfields in which DL and AR are making a fundamental contribution. The potential of DL in intra-operative surgery is formidable: by learning from data of past procedures, it would be possible to monitor the progress of a surgical procedure [10, 11], estimate the pose of the medical staff [12, 13], evaluate skill assessment and provide new ways of interacting with multimodal data during and after a procedure [14], just to name a few examples. All these procedures necessitate an intuitive interface for surgeons and specialist, that could be given by AR applications. Lately, the efficacy of AR as a tool to improve the transfer of information has been the topic of many different research works. In particular, give the surgeon the ability to see what cannot be normally perceived, as the hidden organs inside the body of a patient, is the main addition that AR brings to surgery [15]. Unfortunately, in intra-operative surgery, a joint utilization of DL and AR is still underrated, due to the difficult compromise between working with 3D data and the efficiency needed for real-time elaboration. In a recent paper [16], we outlined a definition of an Intelligent Operating Room (IOR), a collaborative operating room based on highly intuitive, natural and multimodal interaction. In this study, we moved the first step in this context, as we aimed to demonstrate how to apply DL to improve the performances of a previously proposed method from our group [17]. In the former work, resumed in the next section, we integrated different solutions based on localization and 3D augmentation, each dedicated to a specific stage of a prostatectomy, into a single software system to give assistance during robot-assisted radical prostatectomies (RARP). The preliminary method used a complex Computer Vision approach to localize different areas in the endoscopic video. Here, an encoder-decoder structure based on Convolutional Neural Network (CNN) is applied to obtain real-time semantic segmentation of the scene and improve in precision the subsequent 3D enhancement. We compared different combination of segmentation architecture and base neural network to select the most performing one, based on training and testing with 4 different videos (*A, B, C* and *D*) presenting the last phase of prostatectomy procedures. Finally, we used video *E* to validate the performances of the 3D overlay method with a new dataset. The developed application is currently being used during in-vivo surgery, for extensive testing, by the Urology unity of the San Luigi Hospital, in Orbassano (To) Italy, and the augmented video stream can be accessed directly into the Tile-Pro visualization system of the Da Vinci surgical console. The main contribution of this paper is to present a solid evaluation of our methodology to visual overlay a 3D model over a 2D endoscopic stream. The focus of this research is to provide a robust clinical application solution to the stated problem, based on numerical evaluation. Rather than techniques-based solely on Computer Vision (CV) to determine the 3D organ model position and rotation, as in our previous approach [17], we leveraged on the output of a state of the art CNN segmentation architecture, obtaining better results, as shown in “Formulation of the problem” Section.

## Formulation of the problem

In recent years, the adoption of minimally invasive surgery (MIS) technology has grown exponentially, to reduce access to wound trauma and decrease the incidence of post-operative complications [18]. In particular, the high demand for greater surgical precision has led to the birth of robotic surgery. The introduction of surgical robots has given many advantages to surgeons, for example, improved stereoscopic visualization, removal of hand tremors and greater precision and enhanced maneuvering of the surgical tools. In the context of urology, robotic surgery was introduced 15 years ago and is now used worldwide [19] and in particular in the context of radical prostatectomy. Despite the above-mentioned positive improvements, it still presents challenges that require to be solved. Among them, we addressed the problem of the limited field of view offered by the endoscope used for navigation. AR could be the solution to overcome this obstacle, as it combines images from the real world with others digitally produced, with the intent of increasing the information the viewer can obtain from their combination [20]. In particular, when AR is applied to MIS the goal is fusing 3D objects produced from pre-operatory patient data with real-time images taken by the endoscope camera [21]. The challenge then became how to correctly align the virtual objects with their real world equivalent. In a previous work from our research group [17], we presented our progresses in enhancing endoscope video during RARP, by superimposing the 3D virtual model of the patient’s prostate on its 2D counterpart, using different real-time techniques. The proposed framework was divided into 5 main phases that characterize a prostatectomy procedure [22]. To perform the task, a virtual model was generated from high-resolution preoperative medical imaging techniques, such as MRI. This 3D reconstruction accurately reproduces the organ and the surrounding structures of the patient undergoing the operation and was modeled by bioengineers using the HA3D^TM^ technique. According to [22], the steps of this particular procedure are highly standardized, hence we have grouped them into 5 subsequent steps, based on similar visual characteristics and similar levels of benefit from the use of RARP. These 5 steps are: (1) Defatting and incision of the endopelvic fascia. (2) Management of the bladder neck. (3) Vase clamping and nerve-sparing. (4) Surgery by the prostatic apex. (5) Targeted biopsy.

In the 2nd step, the 3D overlay was rarely requested by the surgeon. In the 4th step, 3D reconstruction from MRI is considered not accurate enough in depicting the apex. Hence, we excluded these stages from those requiring AR implementation. Instead, during the 1st step, it is critical for the surgeon to correctly identify and locate the neck of the prostate and in the 3rd step preserving nerves' functionality after the procedure. In these stages, the presence of the 3D model correctly representing organs boundaries was requested by the surgeons to increase their intra-operative perception. Aligning the 3D model of the prostate with its physical counterpart, without clear visual references, proved to be extremely inaccurate. For this reason, we focused on the 5th phase. During this stage, the insertion of a catheter into the pelvic cavity provides an artificial feature easy to be identified and used to guide the virtual-over-real overlay. Superimposing a 3D model along with the cancer position is fundamental to improve the localization of the tissue sample for post-operative biopsies. Our previous approach to detect the catheter leveraged solely on classic CV techniques which made it very fast and suitable to operate in real-time; nevertheless, this method experienced great variability depending on conditions, such as illumination changes or camera movements, resulting in non-optimal performances. We now changed our approach, evaluating the ability of different neural networks architectures to perform semantic segmentation and localize the catheter in the video stream, maintaining an acceptable elaboration speed.

## Methods

### Semantic segmentation

The semantic segmentation of an image aims to associate each pixel with a class of a predefined set. Segmentation techniques are mostly based on CNN. The typical architecture adopted in these procedures uses two opposite branches of CNN and is called the encoder-decoder structure. The encoder has the typical structure of a CNN with convolutional and pooling layers that samples the input to generate a high dimensional characteristics vector. The decoder has the opposite structure: it takes as input a high dimensional characteristics vector and generates a semantic segmentation mask. We explored the best combination of architecture and neural network and selected the most performing one. We tested 3 segmentation architectures among the vast amount of versions: SegNet [23], U-Net [24] and PSPNet [25]. They were chosen for a particular reason related to our objective, as SegNet is specifically designed for real-time task, U-Net was created especially for biomedical imaging, and for this reason, it works very well with a limited number of images, and PSPNet is optimized to learn better global context representation of a scene. The next task was to choose an appropriate base network. The models we decided to test are ResNet [26], VGG [27] and MobileNet [28]. We trained and tested each possible combination of these segmentation architectures and the base networks, except for the combination of PSPNet and MobileNet that was not present in the library used.

### Dataset

**Fig. 1:**
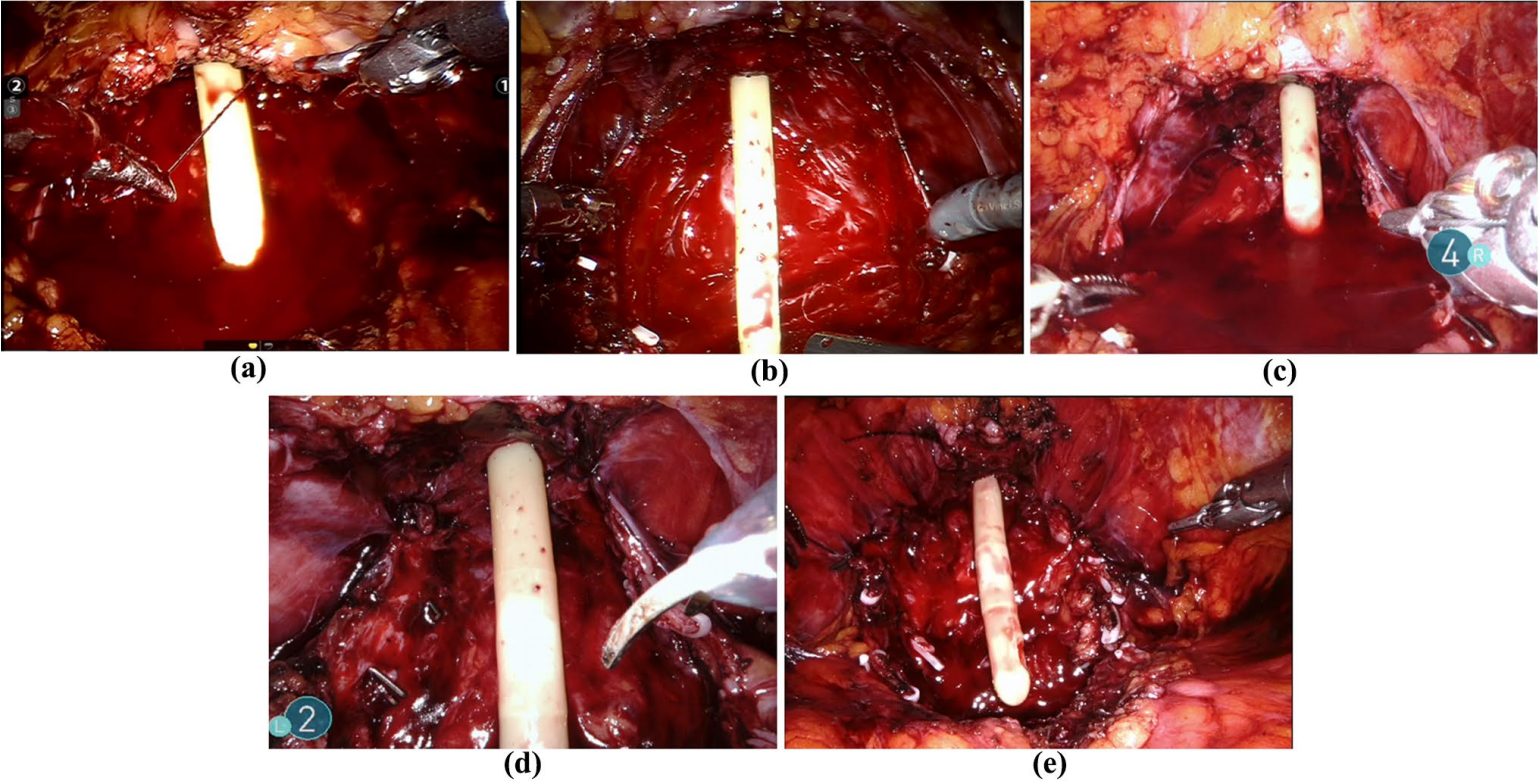
Samples taken from video A, B, C, D and E videos

We collected 5 videos from different surgical procedures showing the insertion of the catheter, i.e., the above-mentioned fifth phase “targeted biopsy”, and we extracted, tagged and resized a set of frames. The first video selected, named *A*, due to its variety of operations and camera-views, and to the fact that the surgical equipment appears and disappears in a continuous fashion, was considered the most exemplary. Adding up the total number of frames of all the videos of different duration, we ended up with approximately 15.570 images. From these, we extracted from *A* 275 images to be used or training plus another 50 images taken from the same video to test the segmentation. Then, 90 images from *B, C* and *D* were selected to try to generalize the results: 50 were used to test the former model, 40 to re-train the model including frames from different videos, after the selection of the best combination of segmentation architecture and encoder network. We decided to use a small percentage of the total number of images to demonstrate that this approach could rely on a limited number of training samples. Each image was manually tagged by two senior urologists using *labelme* [29], an open-source software that provides an interactive GUI to produce the ground truth segmentation for 3 categories: *background, tool* and *catheter*. Nevertheless, we gave importance to optimize the value of the *catheter*, as we needed its location to map the 3D model to the 2D video. Finally, we extracted 100 frames from video *E* to test the overlay performances of the AR framework. Each frame has been selected for its peculiarity (e.g., extreme rotations, partially hidden catheter, uncommon catheter rotational values, etc.) and tagged by our specialists’ team with optimal value of the anchor point (*p1, p2*) and the rotation along the *X*- and *Z*-axis for the 3D model. In Fig.1, are shown some samples for the 5 different videos. All the samples have been resized $416~ \times ~608$ pixels.

### Metrics

Different combinations of network and basic architecture were tested, and the best pair was selected using Intersection over Unit (IoU) metrics and the number of iterations per second (*it/s*). The first metric was used to measure the precision of the segmentation, the second metric to calculate the number of frames that could be processed every second. The IoU is defined as:

$$ {\text{IoU}} = \frac{{AOverlap}}{{AUnion}} $$

where *AOverlap* is the area of overlap between the expected segmentation and ground truth, and *AUnion* is the area of union between the expected segmentation and ground truth. This metric is normalized in the interval [0, 1], with 0 meaning that there is no overlap and 1 meaning a perfectly superimposed segmentation. Each pixel of the network’s output is compared with the corresponding pixel in the ground truth segmentation image. We not only computed the IoU for the *catheter,* but also the Mean IoU, that is the average IoU between the 3 classes. This metric was used to demonstrate that the network was learning to also segment the *tool* and *background* classes. The second metric, *it/s*, is defined as:

$$ it/s = \frac{n}{{\sec }}~ $$

where *n* is the number of iterations, and sec is the time unit. In this case, one iteration consists of predicting the segmentation output given an input image. As the frame rate metric depends on different aspects, such as the complexity of the mesh, the rendering engine used, the hardware specifics, the particular implementation of the pipeline, etc., these factors can determine a strong fluctuation of the metric, for this reason we preferred to opt for a metric independent of these parameters. We empirically noticed that our frame rate was acceptable if we kept the *it/s* greater than 10; this evaluation is not indicative but sufficient for us to obtain a real-time validation*.* Regarding the validation of the 3D overlay, we computed two different metrics. The 3D model is attached to the video stream given the coordinates $\left( {p1,~p2} \right)$ of the anchor point and the rotation along the *X*- and *Z*-axis. The difference between the predicted $\left( {\widehat{{p1}},~\widehat{{p2}}} \right)$ and the actual anchor point $\left( {p1,~p2} \right)$ was evaluated with the classical Euclidean distance between two 2D points: $eucDist = ~\sqrt {\left( {p1 - \widehat{{p1}}} \right)^{2}  + ~\left( {p2 - \widehat{{p2}}} \right)^{2} } .$

The difference between the rotations was computed converting the values of the rotations (considering the one along the *Y*-axis equal to 0, since rarely involved) in quaternions and then calculating the geodesic distance between two quaternion’s coordinates *q1* and *q2.* To get a distance between two unit quaternions, you have to rotate both of them such that one of them becomes the identity element. To do this for our pair *q1* and *q2*, we simply multiplied *q1* by *q2*’s inverse from the left

$$ Q = \left( {inverse\left( {q2} \right)*q1} \right) $$

and normalize the obtained quaternion *Q* through L2 normalization:

$$ geoDist = L2\left( Q \right) = ~\sqrt {Q \cdot Q} $$

The metric is a positive amount corresponding to the length of the geodesic arc connecting *q1* to *q2*.

### Post-processing

From the segmentation output, we used the algorithm proposed by Suzuki et al. [30] to obtain the segmentation contours and the Sklansky’s algorithm [31] to obtain the convex hull given the main contour, to extract the information needed to correctly align the 3D model to the real-time video. This information is:

1. The rotation angles for *X* and *Z*-axis. *Y*-axis is not considered since rarely involved;
2. The anchor point where to plot the upper extremity of the catheter.

**Fig. 2:**
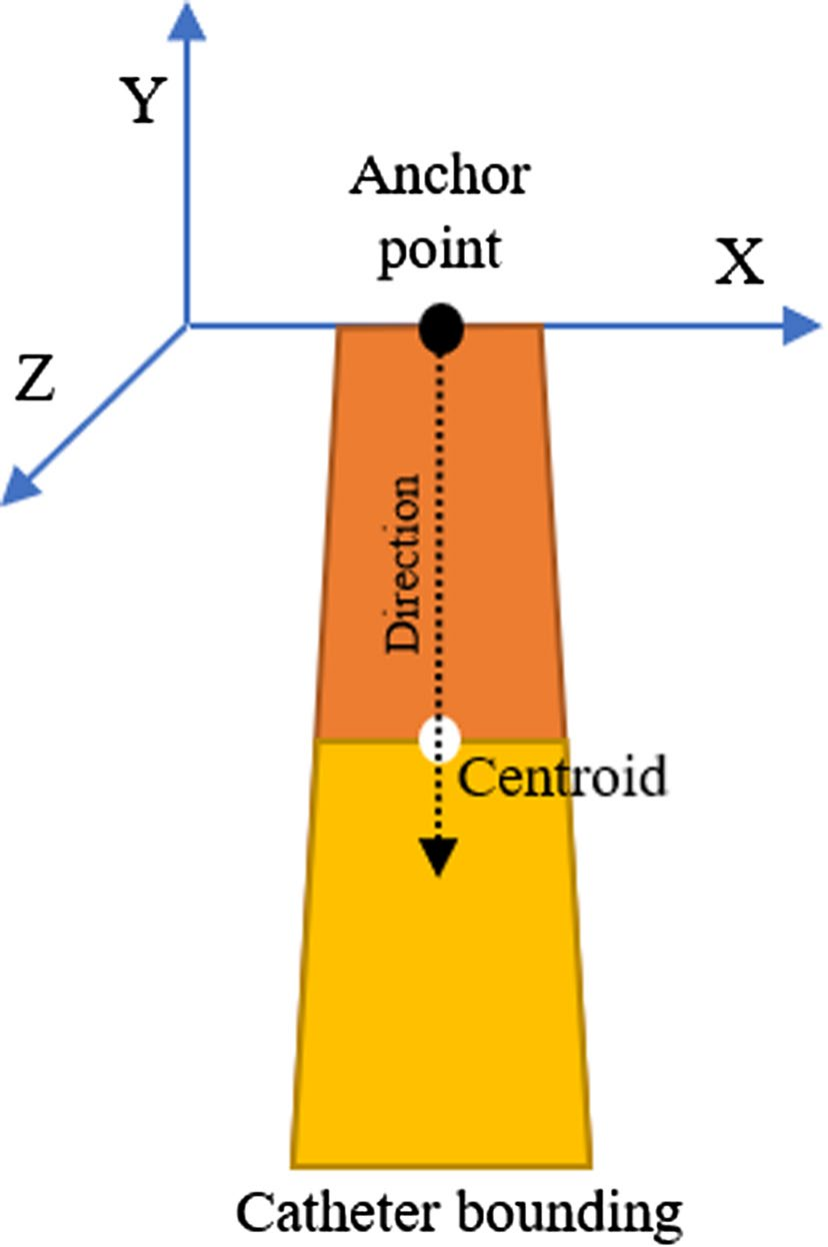
The bounding polygon of the catheter. The upper black dot marks the anchor point, while the white one marks the centroid. The *direction* arrow was used to calculate the *Z* rotation, and the two sections of the area divided by the centroid to compute the *X* rotation

**Fig. 3:**
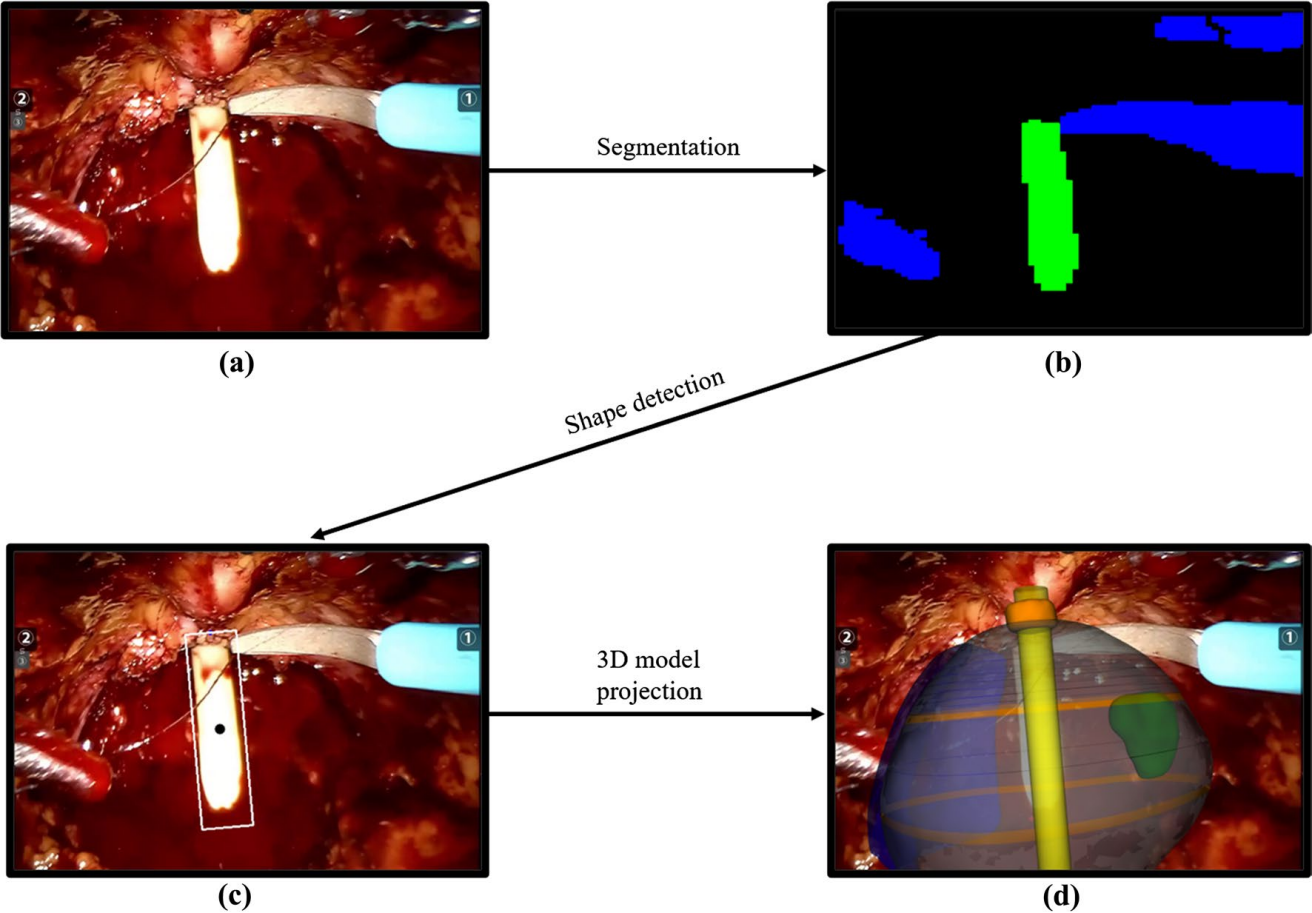
The full pipeline of our method. The original image **a** is segmented **b** and, after the shape detection **c**, the 3D model is projected over the 2D image **d**

In particular, as shown in more detail in Fig. 2, the central point of the upper edge of the boundary box shown as a black dot, is considered the anchor point of the 3D mesh, corresponding to the apex of the catheter. The dimension of the upper edge is compared to the diameter of the catheter in the real world to determine the scale of the virtual model. The vector from the anchor point to the centroid shown as a white dot in Fig. 2, is used to calculate the rotation of the mesh along its *Z*-axis (standard Cartesian coordinate system). The rotation along the *X*-axis is processed by comparing the upper and lower part, divided by the centroid, of the area of the catheter’s shape detected. These two sections are highlighted in Fig. 2 with two different colors. When the lower area is larger than the upper area, the mesh must be rotated toward the camera. When the lower area is smaller than the upper area, which happens less often, the 3D model should be turned so that its base is further away from the camera. The full pipeline of this method is shown in Fig. 3.

### Training, framework, and evaluation

We first trained the model with 275 images of the *A* dataset, tested it with 50 images taken from *A* and subsequently with 50 images from *B, C* and *D* each, to select the best combination of segmentation architecture and CNN for this specific case. Then, we trained the model selected with *A* video plus two among the *B, C* and *D* videos and tested with the excluded one, to obtain more generalized results. This was done with 40 previously unseen images for class *B, C* and *D*. Finally, we trained and tested the model with all the videos. We then take the model trained with *A, B, C* and *D* and tested the overlay precision with 100 frames extracted from video *E*. After trying different configurations, we obtained the best results using a batch size of 4 and Adam [32] optimizer with a learning rate of 0.001 and beta values of 0.9 and 0.999, respectively. The function to calculate the loss was the categorical cross-entropy. We run the model for just 20 epochs before it starts to converge. We used Keras [33], an open-source neural network library written in Python, running on top of TensorFlow, and, in particular, the *keras-segmentation* [34] library, on Windows 10 Pro with NVIDIA Quadro P4000.

## Results

We firstly run all the possible combination between the 3 segmentation architectures (U-Net, SegNet, PSPNet) and the 3 base networks (MobileNet, VGG, ResNet), except the ensemble of PSPNet and MobileNet due to the missing implementation in the Python library. The IoU value obtained is shown in Table 1, for networks trained just with the *A* video and tested with different frames taken from *A, B, C* and *D* videos. The values of Mean IoU are shown for completion, to demonstrate that the network was learning to also segment the *tool* and *background* classes. In Table 1, it is also shown the iterations per second for each combination, because we are working with a real-time application, and we needed the application to have an acceptable frame rate.

**Table 1:** Results training with *A* and testing with A, *B, C* and *D*

Base network	Architecture									
	Test Video	U-Net			SegNet			PspNet		
		Cat IoU	Mean IoU	*It/s*	Cat IoU	Mean IoU	*It/s*	Cat IoU	Mean IoU	*It/s*
MobileNet	A	0.926	0.927	11.04	0.913	0.911	10.96	NA	NA	NA
	B	0.888	0.819		0.763	0.776		NA	NA	
	C	0.467	0.705		0.259	0.611		NA	NA	
	D	0.868	0.753		0.634	0.690		NA	NA	
VGG	A	0.815	0.720	7.81	0.914	0.887	8.02	0.863	0.870	8.68
	B	0.752	0.733		0.846	0.830		0.709	0.736	
	C	0.147	0.537		0.499	0.710		0.324	0.587	
	D	0.580	0.680		0.836	0.735		0.622	0.614	
ResNet	A	0.937	0.933	6.23	0.829	0.847	8.95	0.901	0.900	6.46
	B	0.925	0.811		0.711	0.714		0.792	0.725	
	C	0.626	0.756		0.254	0.511		0.369	0.628	
	D	0.892	0.760		0.646	0.577		0.787	0.678	

Catheter IoU (Cat IoU) and Mean IoU for the available combination of architecture and base network, training with 235 frames taken from *A* video and testing with 50 frames taken from *A, B, C, D.* It is also shown the number of iterations per second for each combination. One iteration consists in predicting the segmentation output given an input image

After picking U-Net with MobileNet as the most performing ensemble for performance and speed, we re-trained the chosen network with *A* video plus two videos among *B, C, D*, and tested it with the remaining one. These results are shown in Table 2, where we also indicate the results of a network trained with all 4 videos.

**Table 2:** Training and testing of U-Net architecture with MobileNet as the base network to generalize the results

Test	Train							
	A		B		C		D	
	Cat IoU	Mean IoU	Cat IoU	Mean IoU	Cat IoU	Mean IoU	Cat IoU	Mean IoU
*A (Baseline)*	*0.926*	*0.927*	*0.888*	*0.819*	*0.467*	*0.705*	*0.868*	*0.753*
A+B+C							0.899 (↑0.031)	0.864 (↑0.111)
A+B+D					0.661 (↑0.194)	0.783 (↑0.078)		
A+C+D			0.841 (↓0.047)	0.840 (↑0.021)				
A+B+C+D	0.925 (↓0.001)	0.927 (=)	0.936 (↑0.048)	0.921 (↑0.102)	0.762 (↑0.295)	0.858 (↑0.153)	0.945 (↑0.077)	0.937 (↑0.184)

We trained the network with *A* video and two videos among *B, C, D*, and tested it with the remaining one. In the first row, the results for the baseline are reported again to make the comparison valid, and improvements are shown in parenthesis. In the last row, are shown results given from training and testing the network with all the videos

**Fig. 4:**
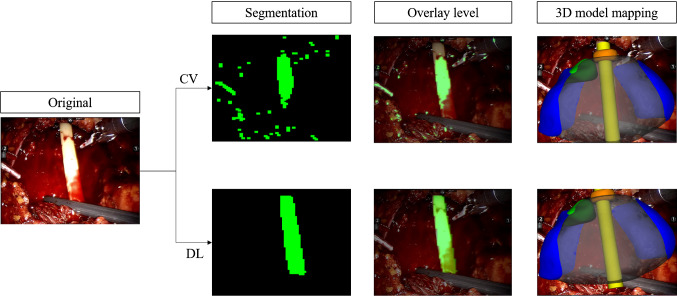
Comparison between the pipeline of the CV-based approach from our previous work [17] and the method proposed on this paper based on DL. It is possible to notice that the approach based on CV predicted a wrong anchor point and slightly worse rotations *X* and *Z*

Then we compared our best network with the one based on Computer Vision techniques used in the previous work. The results of comparison are shown in Table 3 while a graphical example is shown in Fig. 4.

**Table 3:** Comparison between the proposed approach based on Deep Learning (DL) and the former approach based on Computer Vision (CV) techniques for segmentation

	A cat IoU	B cat IoU	C cat IoU	D cat IoU	Mean
DL approach	0.925	0.936	0.762	0.945	0.894 (σ = 0.076)
CV approach [17]	0.450	0.590	0.079	0.240	0.339 ( σ = 0.195)

Standard deviation σ is also shown in the rightmost column

We also evaluated the performances of the 3D overlay with the values of Euclidean Distance between the real and the predicted anchor point and the Geodesic Distance between the *X* and *Z* real and predicted rotations, shown in Table 4.

**Table 4:** Comparison between the proposed approach based on Deep Learning (DL) and the former approach based on Computer Vision (CV) techniques for the 3D overlay performances with standard deviation σ.

	Euclidean Distance	Geodesic Distance	# Images
DL approach	4.160 ( σ = 1.448)	0.169 ( σ = 0.073)	100
CV approach [17]	12.569 ( σ = 4.456)	0.266 ( σ = 0.131)	100

## Discussion

**Fig. 5:**
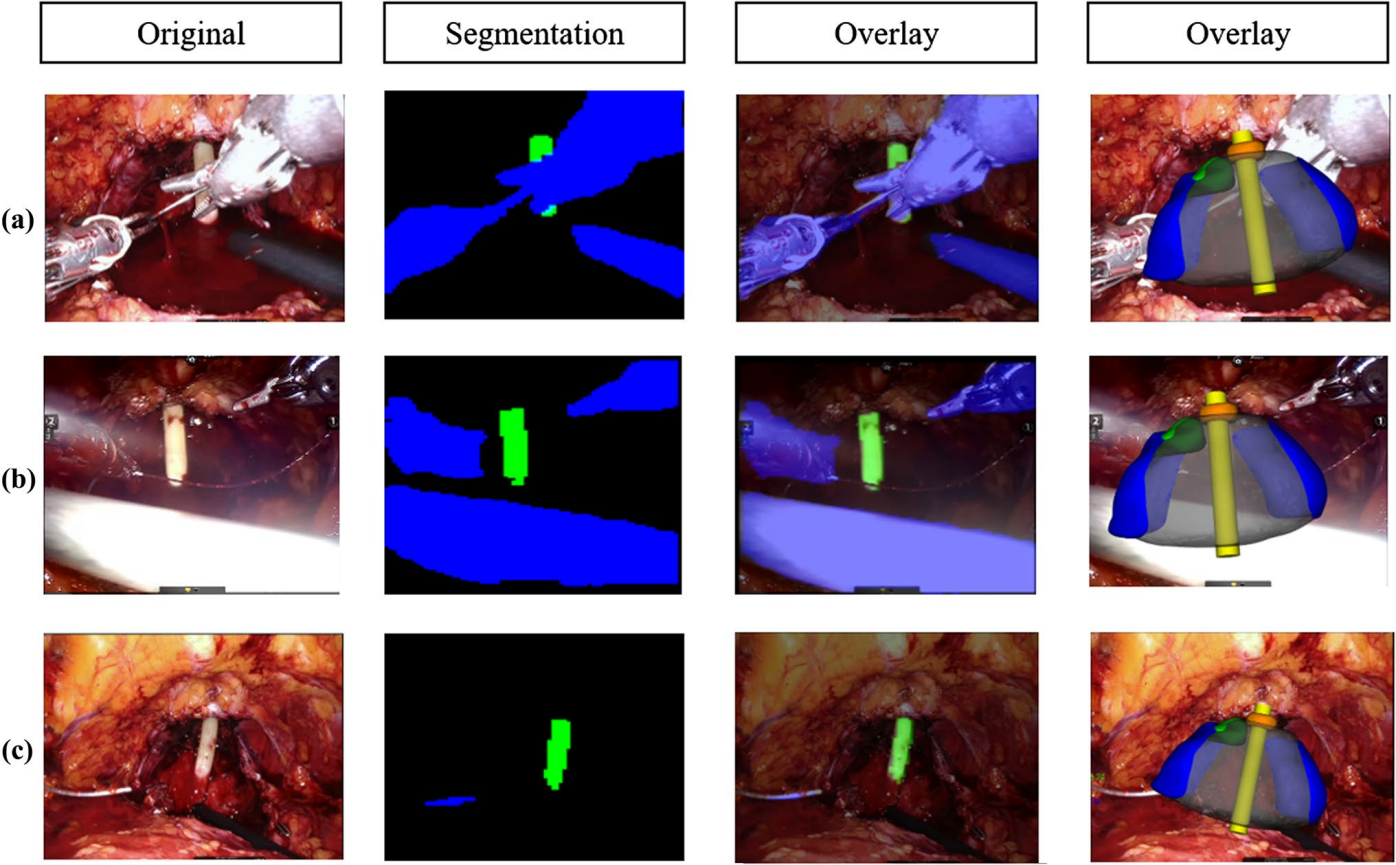
Some segmentation masks returned by our network and the consequent error of the 3D overlay. It is possible to notice how the network performs good even in borderline situations: when the catheter is partially occluded **a**, when a tool is featured in the foreground **b** and when the camera is far from the scene **c**

**Fig. 6:**
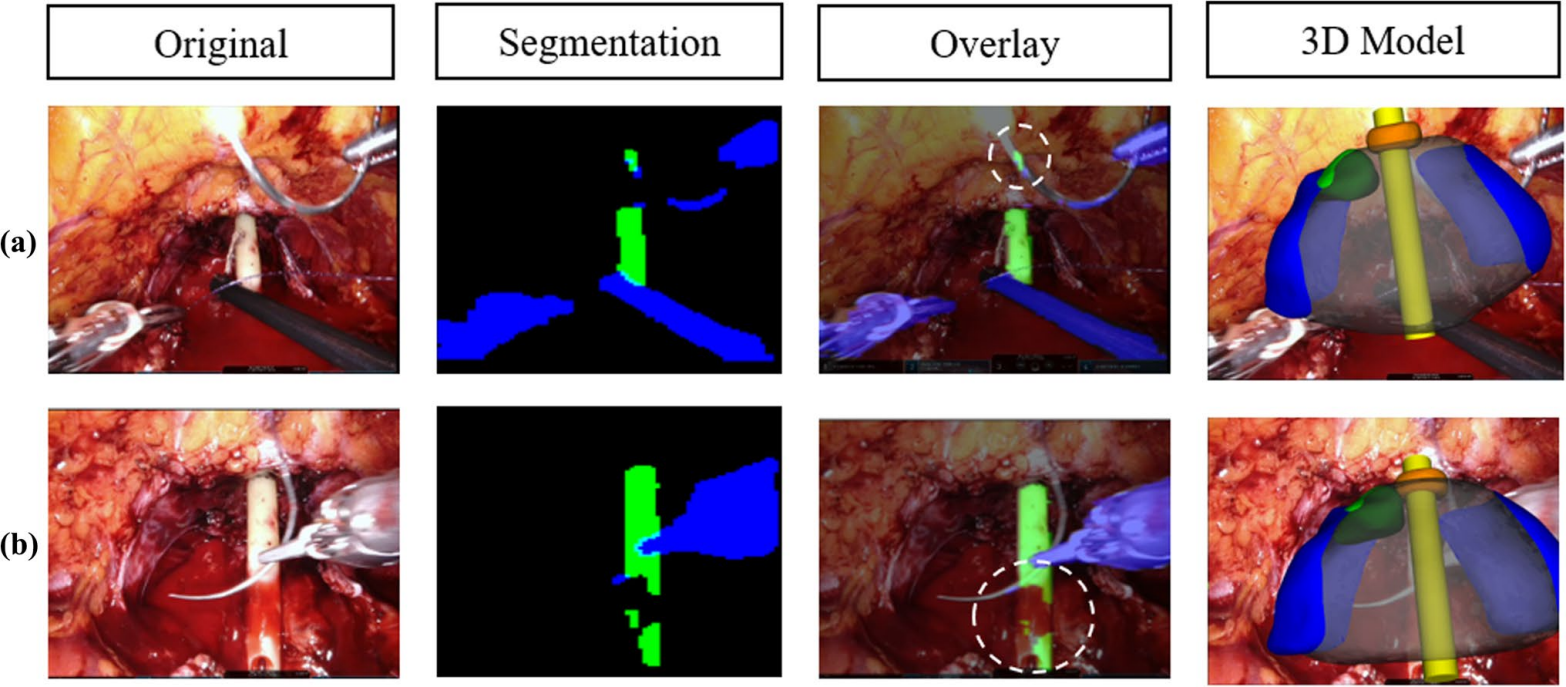
Some examples which shows the errors of the network due to lights **a** and blood presence **b** and the consequent error of the 3D overlay

In this paper, we present the results of our effort to improve the precision of catheter identification in the endoscope frames during the fifth phase of RARP. During this phase, the 3D overlay, based on the correct catheter positioning, is crucial to locate tissue sampling for post-operatory biopsies. For this reason, we trained an encoder-decoder structure for segmentation. Our dataset was composed by frames extracted from 5 different videos: 325 frames from *A*, 90 from *B, C* and *D* each and 100 for *E*. We firstly tested different combination of segmentation architecture and base networks. We performed the training with frames taken from *A* video and the testing with frames from *A, B, C* and *D* videos, and the best catheter’s IoUs were obtained with the U-Net architecture with ResNet and MobileNet as base networks. ResNet reached an average catheter’s IoU of 0.845 while MobileNet 0.780. Nevertheless, MobileNet performed 11.04 *it/s* and ResNet 6.23 *it/s*. As already discussed, we noticed that the frame rate of the real-time application was acceptable if we kept the *it/s* greater than 10: with 11.04 *it/s*, on the hardware used for testing, we are able to reach a frame rate of approximately 8*fps*, which is a good value for a medical application, where the constraint on real-time is not so severe. Since we work with real-time data, we decided to choose MobileNet as a good compromise between precision and speed. We then improved the IoU by training the network with *A* video plus two among *B, C* and *D* and testing with the remaining one. When testing with unseen data, the IoU computed with this approach, had a significant increase, especially for *C* video where the catheter’s IoU passed from 0.467 to 0.661. In the last line of Table 2, we can see the best results. They were obtained when training the network with all 4 videos. These results outperformed the former approach, based on Computer Vision segmentation techniques, where the Gaussian blurred frames were converted to HSV format and binary thresholded given an adaptive pixel range, in order to identify the pixels belonging to the catheter. The average IoU obtained with this former method was 0.339 (*σ* = 0.195) versus an IoU of 0.894 (*σ* = 0.076) of the new proposed approach, as shown in Table 3. After extracting the segmentation mask, we computed the rotation angles and the pivot point to position the 3D model. In Fig. 5, representative samples of the network’s output together with the subsequent 3D overlay are shown. We then evaluated our technique using 100 frames extracted from video *E* and testing the precision of the predicted anchor point and rotation angles, obtaining an Euclidean Distance of 4.160 (*σ* = 1.448) and a Geodesic Distance of 0.169 (*σ* = 0.073), compared to the previous 12.569 (*σ* = 4.456) and 0.266 (*σ* = 0.131), as shown in Table 4. In Fig. 4, a visual comparison of our approach with the previous approach is shown: given a wrong segmentation map, the approach based on CV (1) predicted an anchor point $\left( {\widehat{{p1}},~\widehat{{p2}}} \right)$ with a wrong value of $\widehat{{p2}}$ compared to the actual point $\left( {p1,~p2} \right)$ and (2) returned values of rotations *X* and *Z* slightly wrong, while in our approach it is possible to notice that the value of *X* is correctly rotate the catheter toward the camera and the value of *Z* match the left-right rotation of the catheter. However, there are still challenges to overcome. As shown in Fig. 6, the segmentation network is still vulnerable to abrupt light changes (a) and blood occlusion (b). These issues consequently affect the 3D overlay performances: in Fig. 4 it is possible to notice, also underlined with a dashed white circle, that in (a) the anchor point of the catheter is wrongly detected as the area related to a high presence of light; in (b) the value of rotations are conditioned by the lower part of the catheter, affected by blood occlusion. In future works, we will address this problems by increasing the variability of data samples in the training set acquiring more footage from RARP procedures showing different light and surgical conditions. Concerning the 3D overlay performances, the main problem is related to the anterior posterior rotation along the *Z*-axis, which is often mispredicted. For this reason, we are planning to implement a second CNN to identify the correct *Z* rotation keeping an overall acceptable frame rate.

## Conclusion

In this work, we compared the IoU performances of different neural network architectures, to identify the most precise one to be used for the catheter segmentation. This feature is leveraged on to correctly overlay the 3D prostate model during a specific phase of the robot-assisted radical prostatectomy procedure. We applied an encoder-decoder structure with U-Net as segmentation architecture and MobileNet as base network, the best compromise between precision and speed. We tested the network with different training dataset extracted from different videos to obtain generalizable results. We finally implement a pipeline to map a 3D model to the 2D video stream starting from the output of the segmentation, and compare it with the previous approach-based solely on CV techniques. The former approach was also tested and validated during in-vivo surgery, and the results obtained in the improving of the quality of the biopsy are documented in [17] and [35]. This demonstrated that the post prostatectomy biopsy’s precision increases with the application of our general approach. We are now testing the new improvements introduced in this paper. The evaluation is performed on two equal groups of patients. The first group undergoes biopsy without the aid of the presented system while the second group biopsies were guided by our system. An increased accuracy in biopsies of the second group demonstrates the added value our system is able to provide. The preliminary results are encouraging: of the few cases tested so far with this system, 70% showed an improvement in accuracy in locating the tumor tissue with biopsy thanks to the positioning operated by our system, compared to 50% of the previous approach. The tests will have to be confirmed using a larger number of cases, but we are confident that since in this paper we proved the technical improvement of the overlay precision, and the biopsy is guided by this overlay, the biopsy precision will increase accordingly. In a future publication, we will discuss more precisely the improvements achieved in targeted biopsies using the presented approach, computed with a larger test set of patients. Finally, we will also attempt to extend this approach to other RARP stages with the due adjustments, in order to correctly identify the prostate’s boundaries.
